# Joint associations of 2D–3D urban form with land surface temperature along an urban–rural gradient: Implications for sustainable urban planning

**DOI:** 10.1371/journal.pone.0358985

**Published:** 2026-10-16

**Authors:** Liang Zhang, Xue Liu, Xianfan Shu

**Affiliations:** 1 College of Urban Construction, Zhejiang Shuren University, Hangzhou, China; 2 School of Geographic Sciences, East China Normal University, Shanghai, China; 3 School of Economics, Hangzhou Normal University, Hangzhou, China; Yunnan University, CHINA

## Abstract

As fundamental urban units, buildings are closely associated with urban thermal environments, and their morphological attributes are related to spatial variations in thermal conditions. Although significant progress has been made in characterizing building morphology, the relative importance and joint associations between 2D horizontal characteristics and 3D vertical structures in shaping neighborhood-scale thermal environments along urban-rural gradients remain underexplored. This study integrated multi-source remote-sensing data and Geodetector analysis to examine the joint, grid-level associations of 2D and 3D urban-form indicators with land surface temperature (LST) in Hangzhou, China. Multi-temporal LST data from 2000, 2010, 2015, and 2020 were used to characterize the thermal context, whereas the building-morphology data in 2020 supported a cross-sectional spatial association analysis. The results revealed clear spatial variation in the relative explanatory power of morphological indicators. Specifically, 3D building configurations exhibited the greatest explanatory power in the Core Built-up Area, whereas 2D characteristics showed stronger associations with LST in the Suburban Built-up Area. Interactions between 2D and 3D features generally provided greater explanatory power than individual factors, with several factor pairs showing nonlinear enhancement. These findings highlight the value of jointly considering horizontal land-cover patterns and vertical building form when assessing urban thermal environments. By elucidating gradient-specific thermal regulation processes and multidimensional interactions among built-environment features, this study provides a scientific foundation for spatially targeted, climate-responsive heat mitigation strategies and sustainable urban development.

## 1. Introduction

Urbanization and industrialization are inducing profound landscape transformations, converting natural ecosystems into artificial surfaces dominated by impervious materials [1]. This anthropogenic alteration of the land cover fundamentally modifies surface-atmosphere energy exchanges, leading to the Urban Heat Island (UHI) effect—a phenomenon where urban areas are significantly warmer than their surrounding rural areas [2,3]. Amidst growing concerns over global climate change and the increasing frequency of extreme heat events, understanding and mitigating the UHI effect has become critical for sustainable urban development. The advent of remote sensing technology has enabled the large-scale acquisition of Land Surface Temperature (LST) data, facilitating a crucial shift from qualitative descriptions to quantitative analyses of the urban thermal environment.

Since the UHI effect was first described by Howard in 1833, scholars have conducted a substantial body of research on the formation [4,5], associated factors [6,7], measurement [8,9], simulation [10,11], prediction, and prevention of urban thermal environments [12,13]. It is well-established that factors such as city size, population density, and land-use patterns are closely linked to UHI intensity [14,15]. LST, derived from satellite thermal imagery, has emerged as a primary indicator for quantifying the spatial and temporal dynamics of the urban thermal environment at various scales [16,17]. Analyzing LST on a standardized spatial unit (e.g., a grid cell) facilitates its integration with urban-morphology and socioeconomic data, providing a useful framework for examining factors associated with spatial variation in surface temperature. In parallel, recent advances in high-resolution observation of urban thermal environments have broadened the methodological basis for microscale thermal studies. UAV-based low-altitude multimodal remote sensing can combine thermal-infrared, visible, multispectral, and three-dimensional data, thereby supporting fine-scale characterization of surface radiometric temperature, building morphology, material-related spectral contrasts, shading conditions, and pedestrian-level thermal environments [18,19]. These high-resolution observations complement satellite-derived LST by resolving fine-scale thermal heterogeneity that may be smoothed in medium-resolution imagery, although their spatial coverage, acquisition timing, and sensor-calibration requirements remain more constrained.

The study of the associations between natural, socio-economic, and physical-spatial elements and the urban thermal environment at varying scales exhibits some degree of variability across studies [20–22]. The macro-scale approach is concerned with how population density, city size, spatial structure, and industrial activities relate to the urban thermal environment. The limitations are that the multidimensionality of urban form in the real world is usually ignored and the study areas are mostly developed countries or regions, while there are relatively few cases of cities in developing countries, such as China [23,24]. The mesoscale is mainly centered on the landscape patterns in relation to varying types of land use. However, the spatial associations of landscape pattern composition and configuration with the urban thermal environment vary greatly due to disparate natural elements, urban scale, and urban development stages in different study areas [25]. In general, at the microscale, urban characteristics show a more prominent association with the urban thermal environment [26,27]. Consequently, the examination of the relationship between two-dimensional or three-dimensional patterns of buildings and the urban thermal environment has become a prominent area of research in recent years [28–30]. On the one hand, a significant number of studies have focused on the relationships between two-dimensional morphological patterns and the urban thermal environment, while relatively few studies have addressed three-dimensional morphological features, and their indicator systems are not yet comprehensive; on the other hand, fewer studies have explored the joint associations of the two with the surface temperature. The linkage between urban morphology and the thermal environment does not exist in isolation, as there are extremely complex interactions among the morphological indicators. Previous studies have predominantly employed linear correlation analysis and regression modeling to quantify these relationships, which are insufficient to capture the complex interactions between variables and hinder the understanding of the internal mechanisms.

The urgency of addressing these gaps is underscored by the intensification of summer heatwaves due to global warming, which pose significant risks to public health and economic productivity [31,32]. In recent years, the unprecedented heat events across the Northern Hemisphere serve as a stark reminder of this vulnerability [33]. Hangzhou, a rapidly urbanizing metropolis in China’s Yangtze River Delta, is a representative city grappling with severe summer heat and a worsening UHI effect, fueled by economic growth and population expansion [34]. The city has experienced a marked increase in the frequency and intensity of heatwaves since 2000, with an average temperature rise of 0.4°C per decade [35], making it an ideal case for this research and these results offer valuable insights and serve as a reference for other comparable cities in their exploration of thermal environment mechanisms. This study explores the response of surface and vertical structural features of urban buildings in Hangzhou to LST using a 100m*100m fishnet as the unit of statistical analysis. The objectives of the study are (1) to analyse coupled 2D and 3D architectural features in relation to LST and to explore the relative explanatory power of these variables for LST, and (2) to explore the spatial heterogeneity in multidimensional metrics correspond to surface temperature variations. The results of the study help to elucidate the relationship between Characteristics of Urban Buildings and LST and may have meaningful implications for urban planning and design.

## 2. Materials and methods

### 2.1 Study area

Hangzhou the capital of Zhejiang Province, is located at the southern end of the Yangtze River Delta, approximately 170 kilometers from Shanghai. Its topography is higher in the west, where hills predominate, and lower in the east, where plains and lakes are widespread. The study area (Fig 1) encompasses a predominantly urbanized municipality comprising eight districts: Shangcheng, Gongshu, Xihu, Binjiang, Linping, Qiantang, Xiaoshan, and Yuhang. Baseline mapping of Land Surface Temperature (LST) and 2D/3D morphological indicators was conducted across the entire study area to maintain spatial continuity and provide a holistic overview. To compare building metrics across the urban-rural gradient, the study area was organized into three analytical zones: the Core Built-up Area, the Suburban Built-up Area, and the Peripheral Agricultural and Forestry Zone. This framework reflected Hangzhou’s historical urban structure and observed built-up and land-use patterns; it was not intended to represent statutory planning boundaries. The continuous built-up area across Shangcheng, Gongshu, Xihu, and Binjiang, which form the traditional urban core, was classified as the Core Built-up Area. Relatively concentrated peripheral built-up clusters in Linping, Xiaoshan, Yuhang, and Qiantang were classified as the Suburban Built-up Area; their surrounding areas were predominantly agricultural and forestry land. Areas with sparse or absent buildings were classified as the Peripheral Agricultural and Forestry Zone. The subsequent comparative analysis of associations between LST and building metrics, the gradient comparison is restricted to the two built-up zones, where building footprints were sufficiently widespread for comparable analysis. The Peripheral Agricultural and Forestry Zone was retained in the overall mapping but excluded from the building-metric analysis because sparse building footprints limited the interpretability of building-related statistical relationships.

**Fig 1:**
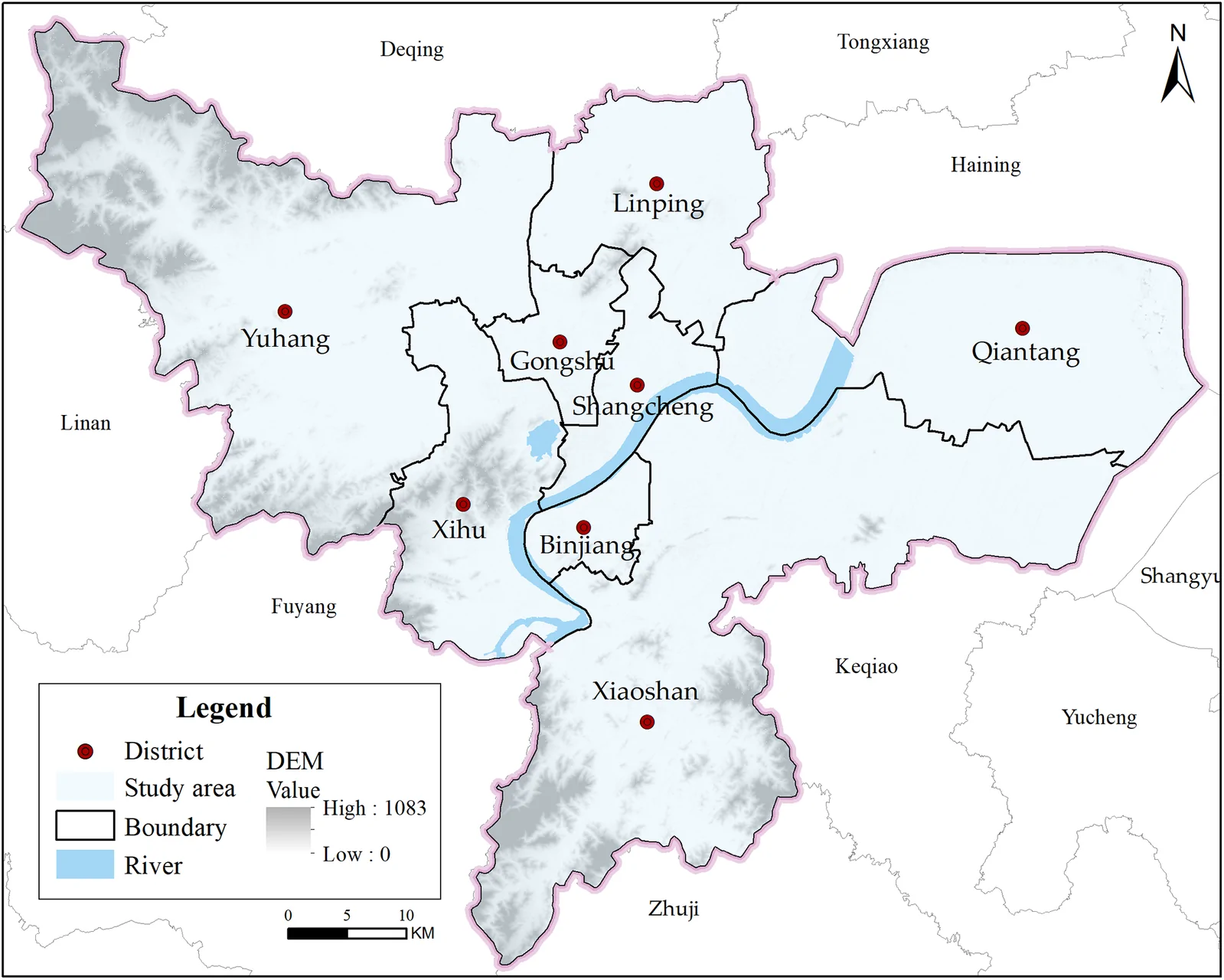
Study area. The basemap data from OpenStreetMap (https://www.openstreetmap.org), and DEM from the Geospatial Data Cloud (https://www.gscloud.cn).

Hangzhou had a built-up area of 615.22 square kilometers. Its urban resident population increased by 3,235,600 with a growth rate of 37.19% during 2010–2020. With socio-economic development and population growth, sustained high temperatures have become more frequent in the city. The maximum temperature reached 41.6°C with 51 hot days in 2013 and 41.8°C during 55 hot days in 2022. Such anomalous thermal events impose substantial challenges on socio-economic activities while posing severe threats to water security, energy supplies, agriculture, and ecological stability. In the context of China’s low-carbon and green transition, growing attention is directed toward the adverse consequences of urban heat, underscoring the imperative to investigate the thermal environment dynamics.

### 2.2 Data and analytical design

The datasets data utilized in this study encompass demographic and economic data derived from statistical yearbooks, meteorological station statistics, geographic information data, building height information data, and remote sensing imagery. Specifically, the temperature data for 2013 and 2022 referenced in the Intro-duction were obtained from the Hangzhou Urban Meteorological Station. Administrative boundaries primarily derive from OpenStreetMap under a CC BY license, with permission from OpenStreetMap Foundation (https://www.openstreetmap.org/copyrigh). The 30m DEM was obtained from the Geospatial Data Cloud (https://www.gscloud.cn). The population and socio-economic data were derived from the Hangzhou Statistical Yearbook 2023 and the Hangzhou Statistics Bureau. Building-height data were obtained from the 10 m Chinese Building Height dataset (CNBH-10m) developed by Wu et al. (2023) and archived on Zenodo (https://zenodo.org/records/7923866) [36]. The CNBH-10m was generated using a Random Forest (RF) model and multisource Earth-observation data, including synthetic aperture radar, optical, and nighttime-light imagery. National-scale validation reported by Wu et al. (2023) yielded a root-mean-square error (RMSE) of 6.1 m, a mean absolute error (MAE) of 5.2 m, and a correlation coefficient (R) of 0.77, indicating reasonable overall agreement between the estimated and reference building heights. The multi-temporal satellite imagery for2000, 2010, 2015, and 2020 were obtained from the USGS Earth Explorer Platform (https://earthexplorer.usgs.gov). To minimize cloud contamination during peak thermal periods, cloud-free Landsat images (< 5% cloud cover) acquired in August or September were selected. Spatial overlay and metric extraction were conducted using standardized 100 m × 100 m grid cells, which served as the fundamental units of analysis and statistical inference. LST and morphological indicators were harmonized to this common grid scale. Accordingly, the estimated relationships characterize spatial associations between LST and aggregated urban-form configurations at the grid-cell scale and should not be directly generalized to individual buildings.

To address the temporal mismatch between the LST and building datasets, a two-part analytical design was implemented. First, multi-temporal LST datasets from 2000, 2010, 2015, and 2020 were employed to provide descriptive temporal context for changes in the regional thermal pattern. Second, because fine-scale building morphology metrics were available exclusively for 2020, the spatial association analysis using Geodetector was limited to 2020. Consequently, the resulting *q*-statistics quantify contemporaneous spatial associations with LST heterogeneity in 2020, and do not assess temporal relationships between building-morphology change and LST change between 2000 and 2020. Administrative districts were used only as geographic reference units for describing the spatial distribution of 2D and 3D building-morphology indicators, they were not used to define the three analytical zones or to represent urban-rural gradient tiers.

### 2.3 Methods

#### 2.3.1 Measurement of land surface temperature.

Landsat remote sensing imagery was utilized to invert surface temperature data. The Radiative Transfer Equation (RTE) method, also known as the atmospheric correction method, is adopted, which is based on the principle of first estimating the atmospheric contribution to surface thermal radiation, subtracting this atmospheric component from the total thermal radiance detected by the satellite sensors, and subsequently converting the resulting radiance into corresponding surface temperature. The surface temperature is obtained by calculating $\varepsilon_{\text{surface }}$ and $B\left(T_{\text{s}}\right)$, which are then calculated from the Planck function.


$$\begin{array}{c} \begin{array}{c} B\left(T_{\text{s}}\right)=\frac{\left[L_{\lambda }-L↑-\tau (\text{1}-\varepsilon )L↓\right]}{\tau \varepsilon } \end{array}\; \end{array}$$ (1)

In Eq. (1), $B\left(T_{\text{s}}\right)$ denotes blackbody-equivalent surface-leaving radiance, and $L_{\lambda }$ denotes at-sensor top-of-atmosphere spectral radiance, which was converted from the digital numbers (DN) of the respective thermal infrared band using sensor-specific radiometric rescaling gains and offsets extracted from the metadata file (_MTL.txt). ε represents surface emissivity; τ is the atmospheric transmittance in the thermal infrared wavelength band; L↑ is the upwelling atmospheric radiance; L↓ is the downwelling atmospheric radiance. The required atmospheric parameters (L↑, L↓, and τ) were obtained separately for each image using NASA’s Atmospheric Correction Parameter Calculator (ACPC; http://atmcorr.gsfc.nasa.gov). ACPC derives them from National Centers for Environmental Prediction (NCEP) atmospheric profiles through MODTRAN-based radiative-transfer simulations. The acquisition date, UTC time, and central coordinates of the study area were entered for each image. Because all images were acquired in summer, scene-specific parameters represented each acquisition without applying a fixed seasonal standard atmosphere.


$$\begin{array}{c} F_{v}=\left(NDVI-{NDVI}_{s}\right)/\left({NDVI}_{v}-{NDVI}_{s}\right) \end{array}$$ (2)

$$\begin{array}{c} NDVI\;=(NIR-R)/(NIR+R) \end{array}$$ (3)

In Eqs. (2) and (3), $F_{\text{v}}$ denotes the fractional vegetation cover; NDVI is the normalized difference vegetation index; ${NDVI}_{s}$ represent the NDVI values of bare-soil, while ${NDVI}_{v}$ represents the NDVI value of fully vegetated endmembers. NIR and R represent the surface reflectance values of the near-infrared and red bands, respectively. For Landsat 5 TM images acquired in 2000 and 2010, Band 4 (0.76–0.90 µm) was used as the NIR band, and Band 3 (0.63–0.69 µm) was used as the red band. For Landsat 8 OLI images acquired in 2015 and 2020, Band 5 (0.85–0.88 µm) and Band 4 (0.64–0.67 µm) were used as the NIR band and red band, respectively.


$$\begin{array}{c} \varepsilon_{\text{surface }}=\text{0}.\text{004}F_{\text{v}}+\text{0}.\text{986} \end{array}$$ (4)

In Eq. (4), $\varepsilon_{\text{surface }}$ refers to the specific radiance of the surface, which describes the efficiency of the land-surface in emitting thermal infrared radiation.


$$\begin{array}{c} \begin{array}{c} T_{\text{s}}=\frac{K_{\text{2}}}{\text{ln}\left[\frac{K_{\text{1}}}{B\left(T_{s}\right)}+\text{1}\right]} \end{array} \end{array}$$ (5)

In Eq. (5), $T_{\text{s}}$ is the temperature of the surface, and $K_{\text{1}}$ and $K_{\text{2}}$ are band-specific thermal calibration constants obtained from metadata file. Landsat 5 TM Band 6 (10.40–12.50 µm) was used for 2000 and 2010. The corresponding constants were $K_{\text{1}}$= 607.76 W/(m²*sr*µm) and $K_{\text{2}}$ = 1260.56 K. Landsat 8 TIRS Band 10 (10.60–11.19 µm) was used for 2015 and 2020. The corresponding constants were $K_{\text{1}}$ = 774.89 W/(m²*sr*µm) and $K_{\text{2}}$ = 1321.08 K.

#### 2.3.2 Indicators for the 2D morphology of buildings.

Buildings represent a primary factor associated with the spatial heterogeneity of the surface thermal environment [37], and building pattern indicators serve as essential tools for quantifying urban building morphology. The study selects indicators from a combination of two- and three-dimensional perspectives to better describe spatial patterns of urban building. The selection of two-dimensional indicators considers the relationship between building site distribution and morphology and land surface temperature. In the rapid urbanization process, the man-made urban landscape dominated by impermeable surfaces gradually replaces the natural landscape dominated by vegetation and permeable surfaces, and the circulation process of water and heat between the surface and the atmosphere changes. Therefore, the proportion and morphological complexity of building land use are closely linked to the urban Land Surface Temperature (LST). Therefore, the study selected the landscape composition indicator of building coverage and two landscape spatial configuration indicators of edge density and the average patch shape indicator (Table 1).

**Table 1:** Landscape indicators for the 2D morphology of buildings.

Indicator	Indicator Description	Calculation formula
BCR	The proportion of built-up land area within the unit	$BCR=a_{i}/A\times \text{1}\text{0}\text{0}$
ED	Edge density within the unit	$ED=\sum_{i=\text{1}}^{n}\;e_{i}/A$
MSI	Building patch shape indicator within the unit	$\begin{array}{c} MSI=\frac{\text{1}}{n}\sum_{i=\text{1}}^{n}\;P_{i}/\left(\text{4}\times \sqrt{a_{i}}\right) \end{array}$

In Table 1, A represents the area of the grid to be analyzed (m^2^); $a_{i}$ denotes the area of category i patches; n is the number of patches of a particular category in the grid to be analyzed; $e_{i}$ is used to denote the sum of the perimeters of the various types of patches within the unit. $P_{i}$ is defined as the mean value of the shape indicator for a specific type of patch.

#### 2.3.3 Indicators for the 3D morphology of buildings.

The three-dimensional indicators were selected mainly from three aspects: height, form, and distribution (Table 2). Average building height (BHM) and height variation (BHSD)are used to reflect the overall height and discrete characteristics of buildings; the average building volume (BMV) and building surface area (BSA) are selected for morphology; and the sky viewability (SVF) and volume ratio (TFAR)are selected for distribution. SVF can synthesize the information of building height and density and characterize the urban landscape morphology more comprehensively, while the basic building indicators such as building height and volume ratio can be directly applied in planning and design, and it is of practical significance to explore their spatial associations with the thermal environment [38,39].

**Table 2:** Indicators for the 3D morphology of buildings.

Indicator		Indicator Description	Calculation formula
Height	BHM	Average height of buildings in the unit	$BH\text{M}=\sum_{i=\text{1}}^{n}\;BH_{i}/n$
	BHSD	Standard deviation of the height of buildings in the unit	$BHSD=\sqrt{\sum_{i=\text{1}}^{n}\;{\left(BH_{i}-BHM\;\right)}^{\text{2}}/n}$
Form	BMV	The average volume of buildings in the unit	$\text{BMV}=\frac{\text{1}}{n}\sum_{i=\text{1}}^{n}\;a_{i}\times {BH}_{i}$
	BSA	Average surface area of buildings in the unit	$\text{BSA}=\frac{\text{1}}{n}\sum_{i=\text{1}}^{n}\;\left(a_{i}+P_{i}\times {BH}_{i}\right)$
Distribution	SVF	Average sky viewability in the unit	$SVF=\sum_{i=\text{1}}^{n}\;SVF_{i}/n$ $SVF_{i}=\text{1}-\sum_{i=\text{1}}^{N}\;{\text{sin}}^{\text{2}}\beta_{i}\left(\frac{\alpha_{i}}{{\text{3}\text{6}\text{0}}^{\circ }}\right)$ N is the total number of sectors in the sky hemisphere that are obscured by obstacles, $\alpha_{i}$ and $\beta_{i}$ are the azimuth and maximum building height angle of each sector, respectively.
	TFAR	The ratio of building area to site size in the unit	$\text{TFAR}=\sum_{i=\text{1}}^{n}\left((\frac{H_{i}}{\text{3}}\;\times a_{i}\right)/A$

In Table 2, n represents the number of patches of a particular category in the grid to be analyzed, $BH_{i}$ denotes the building height of patch i, $H_{i}$ signifies the patch area, $P_{i}$ is the perimeter of patch i, $a_{i}$ denotes the area of category i patches, and A represents the area of the grid to be analyzed.

#### 2.3.4 Detection of explanatory factors.

Regression equations, correlation analysis, least squares regression, and principal component analysis are commonly used to explore the relationship between urban building morphology and temperature, with three-dimensional morphology parameters as the independent variable and measured temperature as the dependent variable. However, most statistical models are based on linear assumptions, which require correlation between variables; and they do not consider spatial autocorrelation, i.e., the interaction between neighboring regions, which makes it difficult to measure the superimposed explanatory power of two variables. Thus, the study adopted Geodetector [40], utilizing its factor detector to quantify the explanatory power (measured by the *q*-statistic) of potential factors regarding the spatial heterogeneity of surface temperature. Concurrently, the interaction detector was employed to evaluate how the coupling of paired variables interactively enhances this explanatory capacity.


$$\begin{array}{c} \begin{array}{c} \;q=\text{1}-\frac{\sum_{h=\text{1}}^{L}\;\;N_{h}\sigma_{h}^{\text{2}}}{N\sigma^{\text{2}}}=\text{1}-\frac{SSW}{SST} \end{array} \end{array}$$ (6)

$$\begin{array}{c} \begin{array}{c} \;SSW=\sum_{h=\text{1}}^{L}\;\;N_{h}\sigma_{h}^{\text{2}},SST=N\sigma^{\text{2}} \end{array} \end{array}$$ (7)

h=1,…,L is the classification of variable Y or factor X; $N_{h}$ and N are the number of cells in the layer h and the whole region, respectively; $\sigma_{h}^{\text{2}}$ and $\sigma^{\text{2}}$ are the variance of Y values in the layer h and the whole region, respectively. SSW and SST are the within-layer sum of squares and the total sum of squares of the whole region, respectively.

The range of value of *q* is [0,1], and the larger the value, the more pronounced the spatial heterogeneity of Y. If the stratification is generated by the independent variable X, the larger the value of *q* is, the stronger the explanatory power of the independent variable X on the attribute Y. When the value of *q* is 1, it indicates that factor X completely controls the spatial distribution of Y, and when the value of *q* is 0, it indicates that factor X has no relationship with Y. The value of *q* indicates that X explains 100 × *q*% of Y. And the interaction between two factors is assessed by comparing the magnitude of *q*(X1∩X2), *q*(X1) and *q*(X2) to evaluate the relative explanatory power of individual and combined factors for the spatial variation of Y. This analysis identifies whether factor interactions exhibit enhanced explanatory power or whether the factors show independent explanatory patterns.

## 3. Results

### 3.1 Temporal and spatial variation of LST

Following the methodology detailed in Section 2.3.1, LST was retrieved for four observation years (2000, 2010, 2015, and 2020). This multi-temporal analysis provided descriptive temporal context for changes in the thermal pattern of the study area. Owing to limitations in the availability of fine-scale building morphology data before 2020, this multi-decadal trajectory was not linked to changes in building morphology and was not included in the subsequent spatial-association analysis in 2020.

The results (Fig 2) demonstrated a gradual upward trend in both the minimum and maximum temperatures observed in Hangzhou between the years 2000 and 2020. Specifically, minimum temperature increased by 76.51%, whereas maximum temperature demonstrated a 23.33% rise. The increase from 2000 to 2010 was significantly greater than that from 2010 to 2020. To ensure interannual comparability, class thresholds were derived from the 2020 LST data using the natural breaks (Jenks) method and then applied consistently to the LST data for all four periods. The LST data were classified into five categories, namely, the extremely high-temperature, high-temperature, moderate-temperature, low-temperature, and extremely low-temperature zones. Between 2000 and 2020, the extremely high- and high-temperature zones underwent the most notable changes. Their proportions increased markedly between 2010 and 2015, from 5.12% and 12.45% to 18.46% and 29.40%, respectively. The maximum proportions were reached in 2015, after which both zones declined. In 2020, the respective proportions were 7.49% and 17.42%. Throughout the study period, the moderate-temperature zone remained relatively stable, whereas the low- and extremely low-temperature zones declined initially and then recovered.

**Fig 2:**
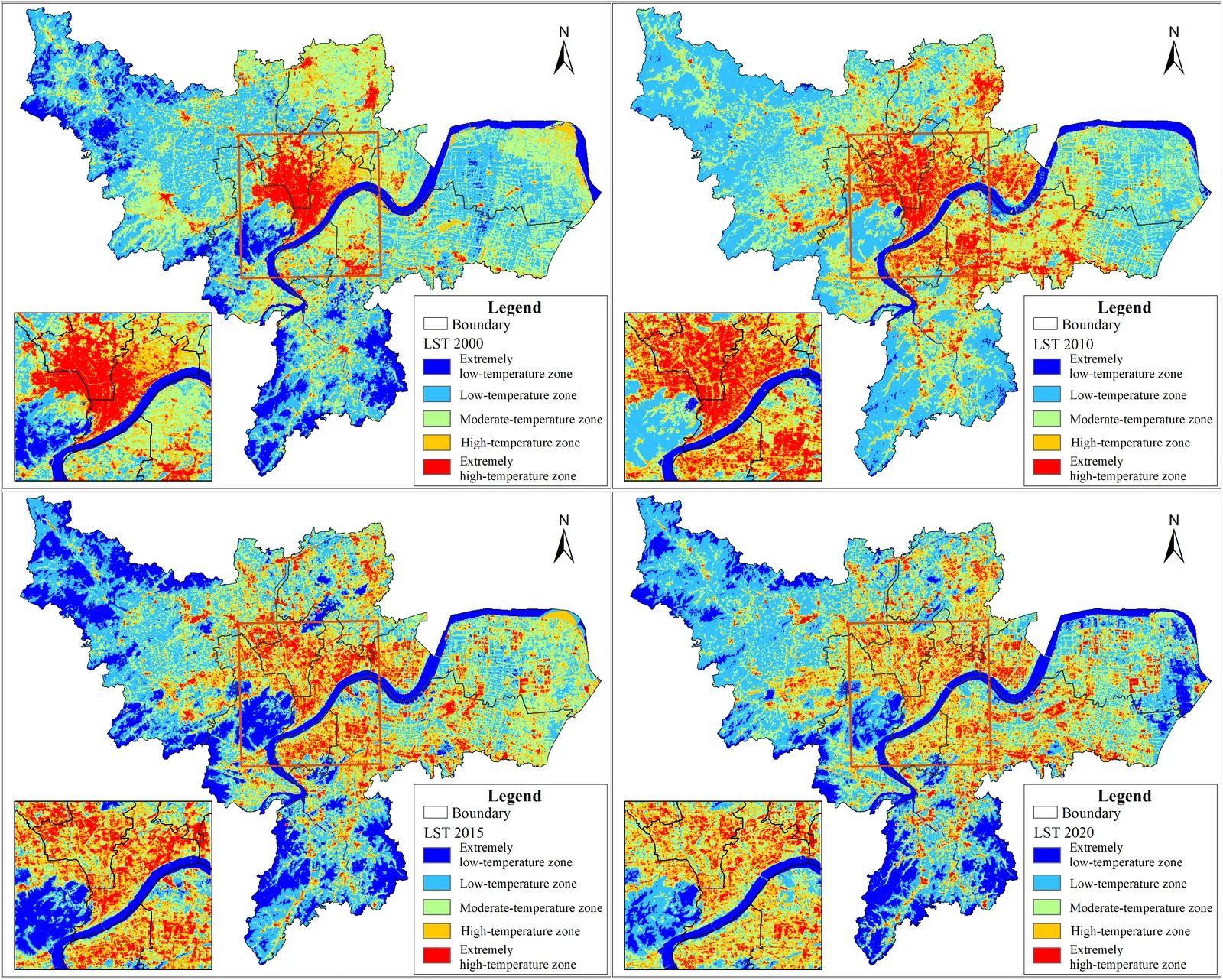
Spatial distribution of LST across four periods (2000,2010.2015,2020). Administrative boundaries from OpenStreetMap (https://www.openstreetmap.org).

From a spatial perspective, the high-temperature regions of Hangzhou displayed a distinct spatial configuration, with a gradual linear expansion from initial aggregation to eventual dispersion. In 2000, spatial disparities in LST were discernible. High-temperature zones were concentrated primarily within the Core Built-up Area, whereas the Suburban Built-up Area and the Peripheral Agricultural and Forestry Zone exhibited relatively low temperatures. In 2010, with the acceleration of urbanization and industrialization, the extremely high- and high-temperature zones underwent a rapid spatial expansion, radiating outward from the Core Built-up Area along primary urban development axes. Consequently, the peripheral very low-temperature zones experienced a notable reduction. Beyond the Core Built-up Area, the high-temperature peak gradually emerged within the Suburban Built-up Area. In contrast to the high-temperature aggregation observed in the Core Built-up Area, the high-temperature areas in 2010 expanded markedly and displayed a decentralized, polycentric spatial pattern. This pattern was particularly evident in the eastern and southeastern parts of the city. During this period, the extremely high-temperature zone within the study area weakened substantially. By 2015, the extremely high- and high-temperatures zones had expanded further, whereas the low-temperature zone had decreased considerably. The extremely low-temperature zone had recovered slightly. Overall, the pattern shifted toward more homogeneous and decentralized coverage. In 2020, both the Core Built-up Area and the Suburban Built-up Area lay within the extremely high- and high-temperature zones and exhibited sustained spatial dispersion and homogenization of the high-temperature range.

The changes in LST were associated with the rapid urbanization in Hangzhou between 2000 and 2020. The gradual expansion of the built-up area, together with increases in urban population and activities, was accompanied by corresponding increases in anthropogenic heat emissions. Concurrently, the increasing diversity and intensity of production and residential activities associated with population growth further increased heat emissions, making the distribution of high-temperature areas broadly consistent with the expansion of urban construction. Areas with intensive production and human activity, such as industrial zones, commercial hubs, tended to have higher surface temperatures. Areas with low surface temperatures were predominantly characterized by well-vegetated mountains and extensive surface-water bodies. Following the construction and enhancement of the green infrastructure and the restoration of the ecological spaces, the proportions of the extremely low- and low-temperature zones increased after 2015.

The multi-temporal LST trend analysis presented in this section provided descriptive, macro-level thermal context for the region; it was not directly coupled with building-morphological changes because historical 3D building datasets were unavailable before 2020.

### 3.2 Characterizing the spatial heterogeneity of multidimensional building characteristic metrics

The measurements for each indicator showed that building morphology in the study area exhibited high spatial heterogeneity (Fig 3). Among the two-dimensional metrics characterizing building spatial form, both ED and BCR showed similar spatial distributions, with high values concentrated mainly within the Core Built-up Area and declining progressively toward the peripheral zones. The BCR of the Core Built-up Area was significantly higher than those of other areas. Moreover, the development of industrial parks and university campuses in the Suburban Built-up Area had contributed to a substantial expansion of built-up areas in this region. High building coverage was concentrated mainly in the historic core sectors and the high-intensity riverside portion (Binjiang), which formed the primary structural backbone of the urban built-up environment. This pattern also reflected Hangzhou’s spatial strategy of shifting from a West Lake-centered development model toward one focused on the Qiantang River. The MSI distribution differed slightly from these patterns, with lower values observed across most areas. However, there are notable exceptions, with Gongshu District exhibiting a higher value for this indicator, followed by Shangcheng District, indicating relatively high building complexity in the central areas of Hangzhou.

**Fig 3:**
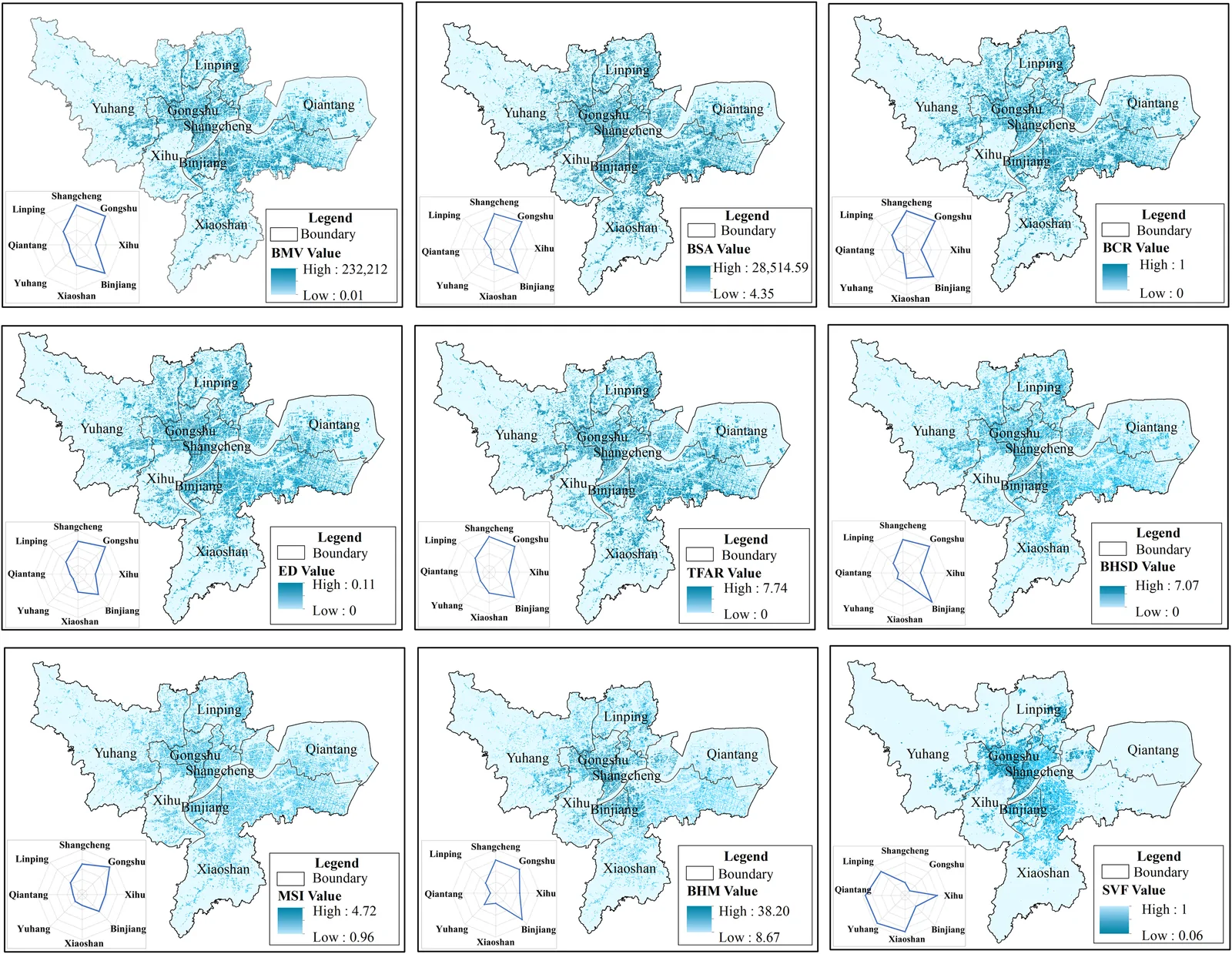
Multidimensional indicators in the study area. Administrative boundaries from OpenStreetMap (https://www.openstreetmap.org).

Among the indicators characterizing the three-dimensional form of buildings, TFAR and BCR reflected the intensity of building development within a given unit. The spatial distribution of TFAR in Hangzhou aligned with that of BCR, showing a high concentration within the Core Built-up Area and a gradual decline toward the suburban and peripheral zones. BHSD and BHM indicated the three-dimensional spatial variation in building form. The BHM distribution pattern followed a low-high-low sequence from the center to the periphery of Hangzhou. In the vicinity of West Lake and the Qiantang River, height controls maintained low-rise traditional structures, whereas surrounding commercial and residential sectors showed a transition toward multistory and high-rise construction. The distribution of BHSD showed markedly elevated values in the riverside portion of the Core Built-up Area, indicating considerable heterogeneity in building heights. The spatial distribution of SVF values, calculated and analyzed using building-raster data, showed low central values and high peripheral values. Lower spatial openness within the Core Built-up Area was associated with reduced solar radiation reaching the surface. Conversely, higher spatial permeability in the peripheral zones is associated with enhanced localized air circulation.

The combination of 2D and 3D indicators showed that urban building patterns were closely linked to the functional characteristics of different urban development contexts (Fig 4). The historic sectors within the Core Built-up Area, primarily Gongshu and Shangcheng, were the most developed and were dominated by residential and traditional commercial uses, resulting in high building coverage. Meanwhile, the area surrounding West Lake has retained its traditional low-rise buildings, which were generally below 10 m in height. The expansion of modern high-rise buildings in adjacent areas, however, produced a more diverse cityscape and increased architectural differentiation. The two banks of the Qiantang River represented areas with the high economic and land values and were dominated by commercial, business, cultural, and leisure uses. Consequently, the riverside portion around Binjiang showed exhibit high building coverage and relatively high values for the three-dimensional height indicators of buildings. It also exhibited the highest degree of building complexity and the most significant variation in building height. Xihu District, which combined higher-education institutions with scenic sites such as Xixi Wetland, showed relatively favorable ecological living conditions, as reflected in its comparatively high SVF. Its BHM and MSI values were slightly lower than those in the other parts of the Core Built-up Area, whereas BCR and TFAR showed a similar pattern. The Suburban Built-up Area, adjacent to the Core Built-up Area, was dominated by dispersed low-rise structures, extensive industrial zones, and continuous logistics parks. Its land-use intensity was lower than that of the Core Built-up Area, and its TFAR was close to that observed in Xihu District. BSA and BV were also comparatively low. This indicated a more decentralized development pattern and outward expansion toward the built-up fringe. Overall, these indicators revealed clear spatial differentiation in building morphology across the study area.

**Fig 4:**
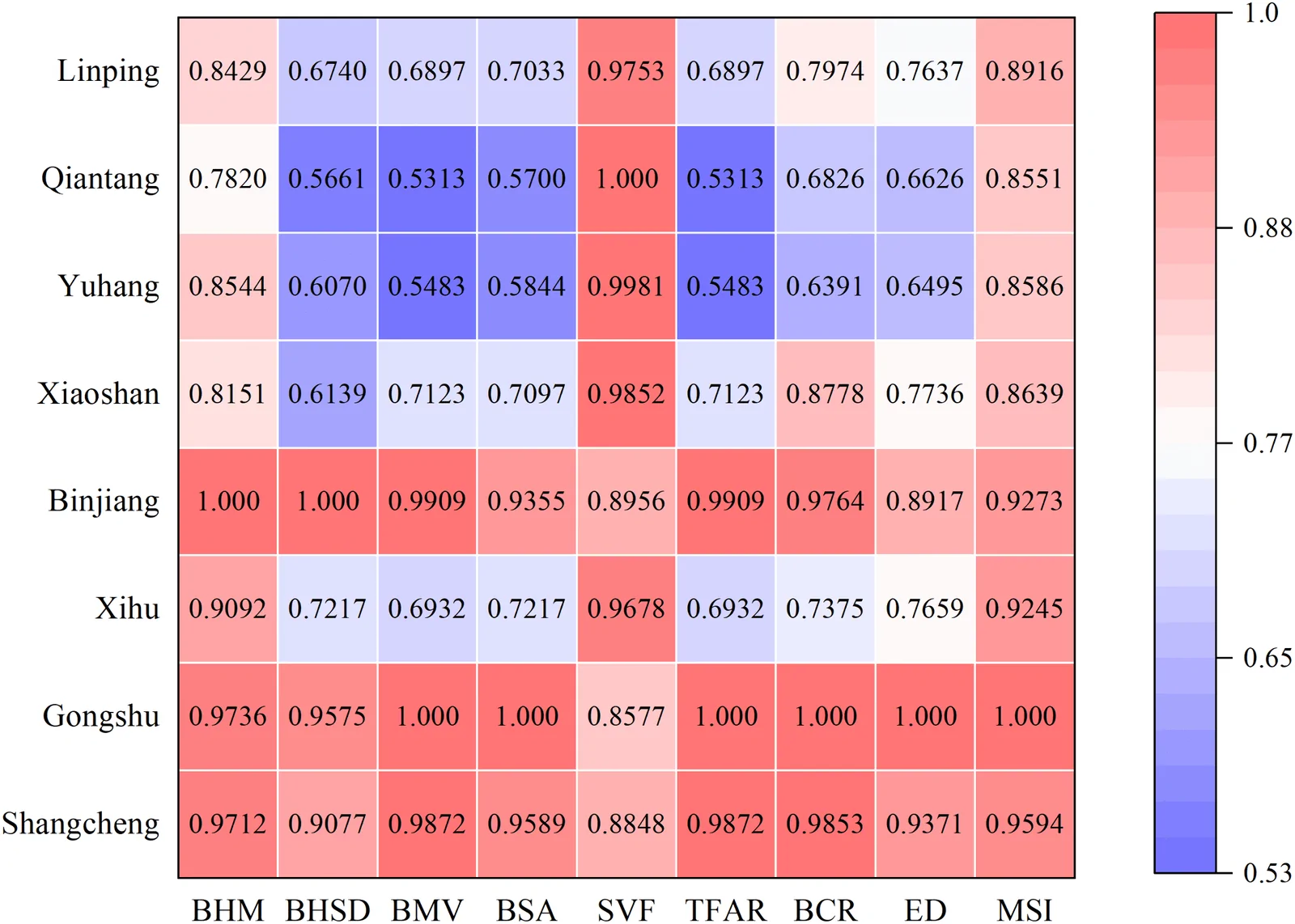
Maximum-normalized indicator values for each district.

### 3.3 Spatial associations between multidimensional building characteristic metrics and LST

The surface temperature data for the year 2020, obtained using Radiative Transfer Equation method, were selected for analysis because they corresponded to the building data for the same year. In accordance with the Geodetector principle, data for the aforementioned indicators, designated as independent variables X1 to X9, were classified into nine categories using K-means clustering in the SPSS software. LST was designated as the dependent variable, Y. Detection results for each variable were obtained by applying the Geodetector method with the (Y, X) input format. As shown in Table 3, the different explanatory variables exhibited varying levels of explanatory power for the spatially stratified heterogeneity of LST, as indicated by their *q*-statistics. The relative explanatory powers of the morphological variables for LST spatial variation were ranked in descending order of the corresponding *q*-statistics as follows: BHM (0.4530) > MSI (0.4281) > ED (0.3974) > BSA (0.3593) > BCR (0.3510) > BHSD (0.3227) > BMV (0.2695) > TFAR (0.2428) > SVF (0.0973). These results indicated that BHM had the strongest explanatory power for the dependent variable, followed by MSI and ED. Furthermore, the results indicate that BSA and BCR also showed substantial explanatory power for surface temperature. The explanatory power of BMV and TFAR had moderate explanatory power, whereas SVF had relatively low explanatory power.

**Table 3:** *q*-value of each explanatory factor.

	BHM	BHSD	BMV	BSA	SVF	TFAR	BCR	ED	MSI
*q*	0.4530	0.3227	0.2695	0.3593	0.0973	0.2428	0.3510	0.3974	0.4281
*p*	<0.001	<0.001	<0.001	<0.001	<0.001	<0.001	<0.001	<0.001	<0.001

With regard to interactions between factor pairs, the results at the level of the overall scale showed enhanced explanatory power, with *q*(X_m_∩X_n_)>MAX(*q*(X_m_), *q*(X_n_)). The factor pairs therefore showed nonlinear enhancement, with their interaction *q*-statistics exceeding the *q*-statistic of either factor alone. The stronger interactions involving building height may have reflected the vertical expansion of urban buildings, which was associated with differences in local- and regional-scale surface-energy balance and airflow. This vertical information provided a more comprehensive characterization of height-related factors and was associated with greater explanatory power for spatial variation in LST. In Table 4, BHM showed the highest interactive explanatory power (0.500) when paired with BCR. This strong association may have reflected a synergistic interaction in which greater building coverage and height coincided with concentrated human activity and energy consumption, which co-varied with higher surface temperature. MSI interacted with all other factors and produced high level of explanatory power, indicating that the shape complexity of the building patches and factors such as BSA, BMV, and TFAR possess significant explanatory power regarding LST heterogeneity. Moreover, interactions between any two factors enhanced explanatory power in a nonlinear manner. Factors with relatively weak individual explanatory power, such as BMV and TFAR, showed greater explanatory power after interacting with other factors. In addition, the 2D indicators showed stronger explanatory power for LST when interacting with the 3D indicators, in some cases exceeding that of BHM. SVF, which had relatively low explanatory power as a single factor, showed more than a twofold increase in explanatory power when interacting with BHM or MSI. These findings indicated that interactions between 2D and 3D indicators provided a stronger framework for explaining spatial variation in LST.

**Table 4:** Results of the interaction detector between two factors.

	BMV	BSA	TFAR	BHM	BHSD	BCR	ED	MSI	SVF
BMV									
BSA	0.3646								
TFAR	0.2704	0.3649							
BHM	0.4952	0.4950	0.4939						
BHSD	0.3749	0.4117	0.3661	0.4662					
BCR	0.3800	0.3964	0.3782	0.5000	0.4175				
ED	0.4073	0.4122	0.4063	0.4884	0.4241	0.4152			
MSI	0.4595	0.4614	0.4582	0.4552	0.4405	0.4640	0.4602		
SVF	0.3546	0.4226	0.3328	0.4912	0.3920	0.4187	0.4471	0.4652	

To evaluate the spatial variation along the urban-rural gradient, the study conducted a detailed comparative analysis between the Core Built-up Area (Zone 1) and the Suburban Built-up Area (Zone 2). The empirical findings presented in Fig 5 indicated that the roles and relative importance of multidimensional landscape- pattern indicators differed substantially across these stratified urban zones. Specifically, within the Core Built-up Area, BHM showed the highest *q*-statistic for LST, followed closely by BCR. This comparative analysis revealed that the association between the three-dimensional spatial configuration of buildings and LST was more pronounced within this dense built-up environment. This urban environment was characterized by prevalent high-rise buildings, extensive building coverage, limited sky openness, and constrained air circulation, with building density and land-use intensity showed relatively strong spatial associations with LST variation.

**Fig 5:**
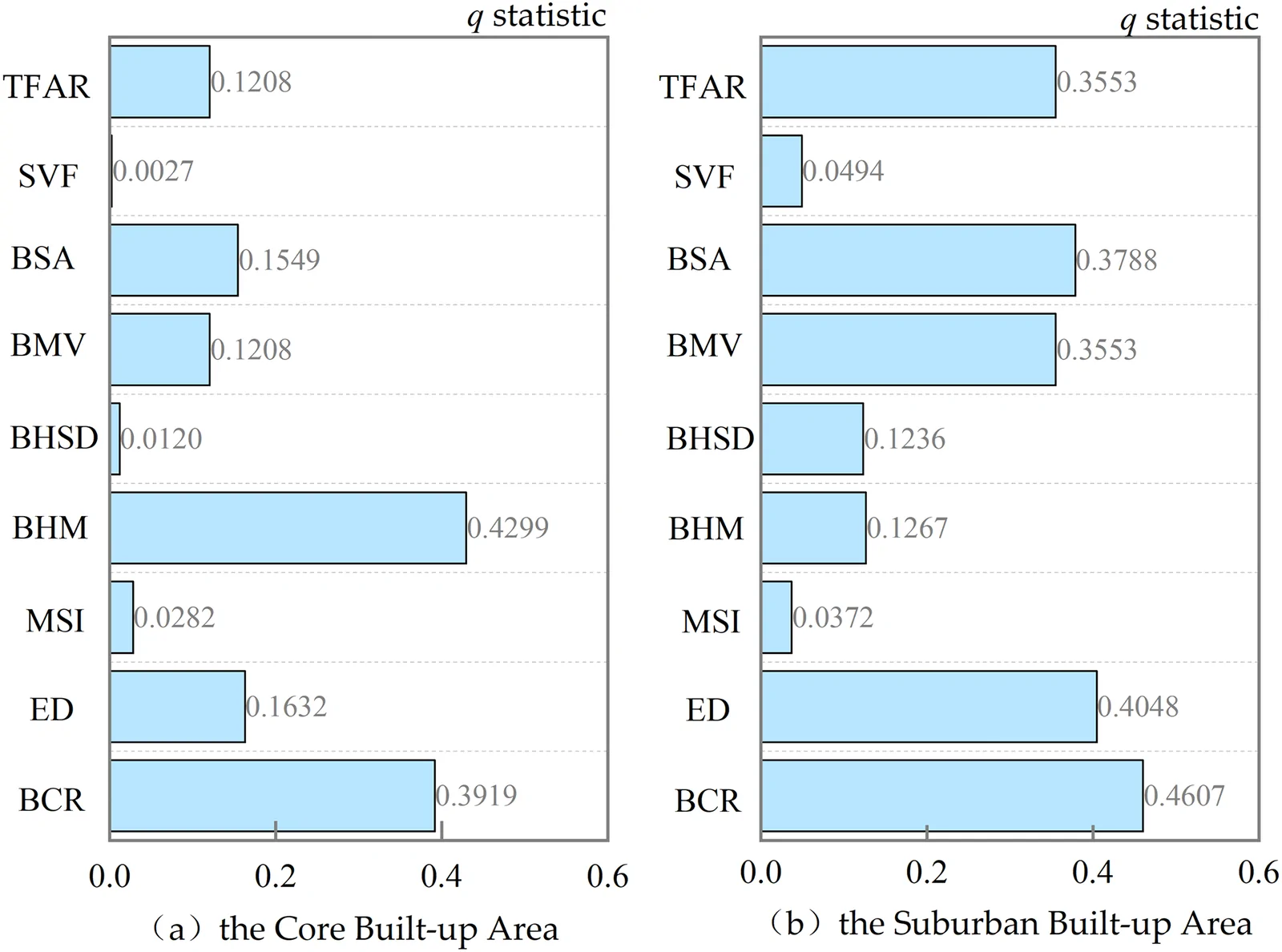
Comparison of *q* values between the Core Built-up Area and the Suburban Built-up Area.

In contrast, within the Suburban Built-up Area, BCR showed the greatest explanatory power, followed by ED and BSA, whereas BHM made a comparatively weaker statistical contribution. This suburban zone was characterized by relatively dispersed human activity, few high-rise buildings and a dispersed building distribution and substantial variation in building height. Consequently, the two-dimensional building-coverage metrics had greater explanatory power for the spatial variation in LST in the Suburban Built-up Area. Moreover, because buildings were lower and more widely spaced in this zone, more thermal radiation may be reflected from buildings to the land surface rather than being trapped in deep urban canyons. Smaller building volumes may be associated with different shading and heat-storage conditions. Under these combined environmental conditions, horizontal configuration indicators such as BCR, ED, and BSA exhibit a much stronger association with LST within the Suburban Built-up Area.

## 4. Discussion

### 4.1 Physical mechanisms underlying multidimensional coupling relationships

The findings indicate that the integration of 2D landscape indicators and 3D morphological indicators exhibits stronger explanatory power for LST than any single factor alone. Most previous studies have focused on comparing the relative significance of 2D versus 3D indicators [41] or examined linkages between 3D morphological indicators and LST [42]. However, evidence regarding their relative contributions remains inconsistent [43]. Some studies have reported that correlations between 2D indicators, such as vegetation cover and building density, and LST are stronger than those observed for many 3D indicators [44]. In this study, 2D indicators showed higher explanatory power overall than 3D indicators, although building height emerged as the primary explanatory factor. On the other hand, although some studies have examined the positive or negative correlation between building height and LST [45,46], they have typically treated it as an isolated factor, neglecting other relevant landscape metrics. Indeed, analysis of the coupling between 2D and 3D indicators revealed a significant strengthening of their joint explanatory power. In this study, the explanatory power of any single factor was generally weaker than that of factor interactions, as illustrated in Fig 6(a). The interaction results further demonstrate that the joint explanatory power of paired factors was significantly enhanced, yielding greater explanatory capacity than when considered individually, as illustrated in Fig 6(b).

**Fig 6:**
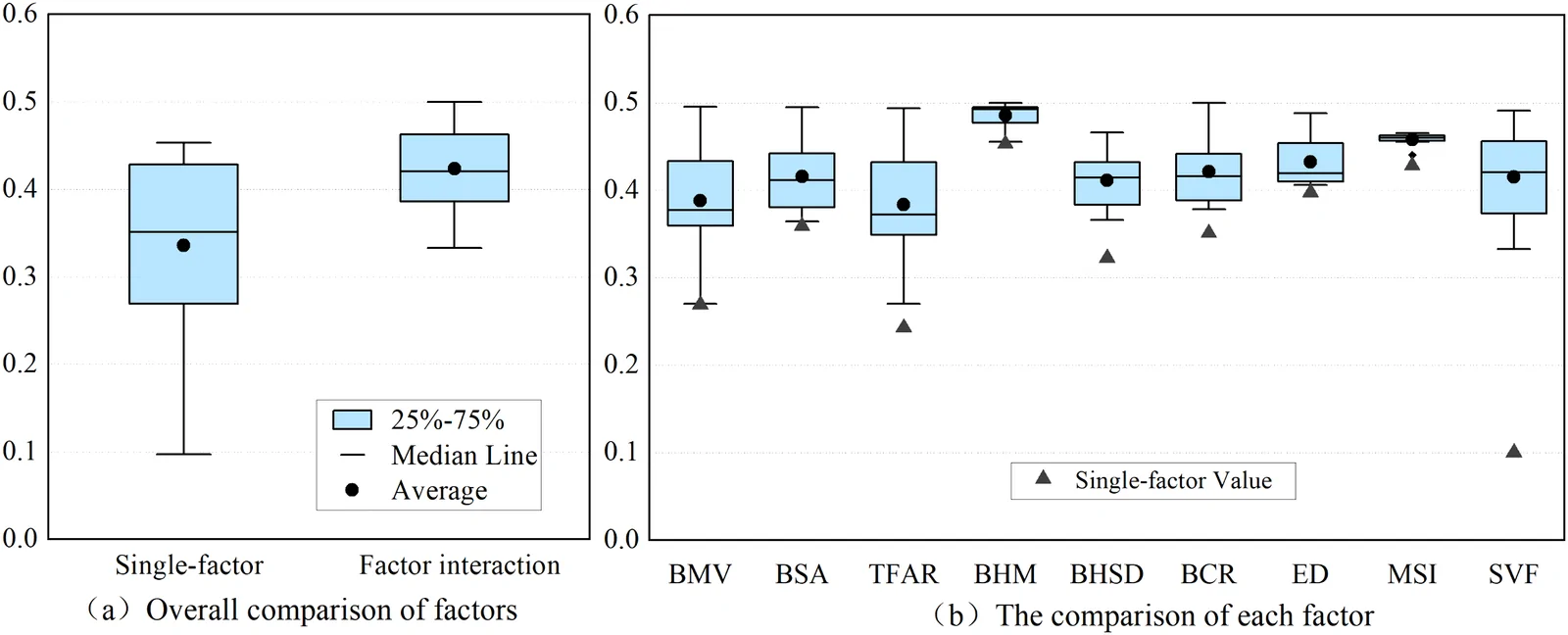
Explanatory power of the single-factor and interaction results.

This enhancement is consistent with coupled controls on the urban surface energy balance [47,48]. Two-dimensional indicators characterize the horizontal distribution of built-up land, vegetation, and exposed surfaces, which correspond to spatial differences in net radiation, evaporative cooling, and surface heat storage [49–51]. Three-dimensional urban configurations may modify sky exposure, mutual shading, longwave radiative exchange, and aerodynamic roughness, thus relating to turbulent heat exchange and local ventilation [38,47,52,53]. BCR represents the proportion of built-up land within a grid cell and thus characterizes horizontal development intensity. BHM represents the average building height within a grid cell and serves as an indicator of vertical development intensity. Because BHM does not directly quantify building spacing, orientation, street geometry, or SVF, its association with shading and airflow should be interpreted as reflecting the broader vertical configuration with which mean building height co-varies. Such coupling may arise because horizontal land-cover composition is associated with variation in radiative loading and heat storage, whereas vertical geometry co-varies with energy interception and dissipation. The enhanced interaction *q*-statistics therefore indicate coupled grid-level spatial associations among radiative, heat-storage, and turbulent-exchange processes; they do not directly estimate physical interaction coefficients [40]. Material thermal admittance may further be associated with variations in heat storage and release, but it was not explicitly measured and is therefore considered a source of residual uncertainty in Section 4.4.

Joint consideration of surface pattern features and building morphology provides a more comprehensive perspective on their combined associations on LST [54]. In real urban environment, horizontal land-cover patterns and vertical building forms interact in a complex manner. For instance, the configuration and elevation of buildings are jointly associated with solar radiation absorption, airflow, and other microclimatic factors. Therefore, integrating building height data with feature indicators derived from 2D pattern parameters enables a more detailed characterization of both horizontal and vertical dimensions of urban form. This approach facilitates a more comprehensive and systematic understanding of the relationship between urban form and LST [8].

### 4.2 The spatial dependence between multidimensional morphometric indicators and LST

The empirical results of this study reveal that the spatial associations between multidimensional morphological indicators and LST vary distinctively across the urban-rural gradient. Within the Core Built-up Area, BHM emerges as the most significant factor explaining LST, whereas BCR possesses higher explanatory power within the Suburban Built-up Area. Previous studies have focused on the relationships between 3D morphological indicators—including SVF, building height, surface area, and volume—and LST across various temporal scales [55,56]. However, our findings indicate that the explanatory power of the examined factors diverges fundamentally depending on the urban spatial scale.

These discrepancies can be attributed to distinct physical configurations and local microclimatic dynamics across urban-rural gradients. Within the Suburban Built-up Area, contemporary urbanization is primarily expressed through horizontal expansion. These peripheral built environments are characterized by sprawling, low-rise building and massive impervious surfaces. In this setting, BCR represents the proportion of a grid cell covered by building footprints and can therefore capture spatial differences in horizontal development intensity. Where the built fabric is relatively low-rise and open, mean building height may exert a weaker association with canyon-related shading and aerodynamic processes. Differences in BCR may then be more closely associated with solar exposure, surface heat storage, and the land area available for vegetation-mediated evaporative cooling [50,51]. Consequently, LST patterns in these suburban sectors are likely to be more strongly associated with 2D horizontal coverage attributes and immediate radiation exposure, while the additional explanatory contribution of 3D volumetric metrics remains constrained [57].

By contrast, the Core Built-up Area is characterized by dense high-rise clusters, whose vertical configurations may be associated with greater mutual shading and lower daytime surface heating. Furthermore, the systematic integration of urban green infrastructure and blue spaces between buildings, combined with the canopy shading of tall buildings, further enhances localized surface cooling and mitigates thermal accumulation [58,59]. The high explanatory power of BHM in the Core Built-up Area is consistent with the prominent association of vertical development with sky exposure, mutual shading, and canyon-scale radiative exchange [47,52]. Higher mean building height may be associated with reduced daytime surface heating where mutual shading is substantial; however, the net thermal outcome also depends on building spacing, orientation, and street geometry, which were not independently quantified in this study. Canyon-related roughness may further be linked to turbulent heat exchange and ventilation. Accordingly, the association between BHM and LST cannot be assumed to be uniformly positive or negative. This interpretation helps explain why height-based indicators may display context-specific associations with LST in dense urban areas [60].

Furthermore, morphology–LST associations may vary across cities because of differences in climate regime, urban form, and development stage. Hangzhou has a humid subtropical monsoon climate with hot, humid summers and episodes of local atmospheric stagnation. High atmospheric humidity may reduce vapour-pressure deficits and constrain evaporative cooling, potentially altering the relative contributions of geometric shading and aerodynamic exchange. Hangzhou also contains a compact high-density core, major natural water bodies and suburban industrial expansion zones. This distinctive spatial configuration, combined with high-rise commercial and residential clusters, may generate localized contrasts in solar exposure, heat storage, and airflow that differ from those in low-density or more regular urban fabrics. Finally, with respect to development stage and spatial governance, Hangzhou represents a fast-growing Chinese megacity in transition from rapid horizontal expansion to intensive urban stock optimization [61]. The high land-use intensity and localized building height regulations govern volume-to-surface area ratios, creating zone-specific thermodynamic signatures potentially associated with local spatial planning policies and rapid post-industrial urban transformations.

Collectively, these insights demonstrate that architectural pattern and spatial morphology are closely related to the localized LST, with the magnitude and direction of this statistical association co-varying systematically with structural complexity across the urban-rural gradient. Characterizing these spatially explicit and context-dependent relationships provides a robust empirical and theoretical foundation for informing climate-resilient urban design and spatially differentiated planning interventions in rapidly expanding megacities.

### 4.3 Practical implications for urban renewal and climate-adaptive planning

The empirical findings show spatially explicit grid-level associations between building morphometrics and LST across the built-up zones, which can inform climate-adaptive planning, urban renewal, and fine-scale urban design while recognising the scale-specific nature of the analysis.

For fine-scale urban renewal within the Core Built-up Area, where 3D morphological metrics showed greater explanatory power, central-city redevelopment may assess opportunities for vertical spatial optimization. Previous evidence from residential areas in Beijing has reported associations between building height and LST [62]. Accordingly, redevelopment proposals may assess compact vertical massing alongside site-specific considerations of mutual shading, building spacing, and ventilation conditions. Planning strategies may consider staggered building heights, appropriate layout density, less complex building massing where feasible, and the preservation of local ventilation corridors while considering shading conditions.

Conversely, for climate-adaptive planning in the Suburban Built-up Area: where 2D coverage metrics showed greater explanatory power, spatial controls may focus on managing building footprint coverage, plot -level density and size [63], and integrating connected blue-green spaces that provide opportunities for shading and evapotranspiration within horizontally expanding built environments.

The coupled multidimensional indicator framework proposed in this study offers a spatial-screening approach for identifying areas where 2D and 3D urban-form attributes may warrant priority consideration in climate-adaptive urban planning.

### 4.4 Limitations and future perspectives

Several potential limitations of this work deserve explicit mention. First, regarding research design and causal inference, the association analysis is inherently constrained by its cross-sectional nature, relying on fine-scale 2D/3D building morphology metrics from a single benchmark year (2020). Although the multi-temporal LST trajectory (2000–2020) establishes a valuable descriptive temporal context for the regional thermal background, historical fine-scale 3D architectural datasets were unavailable for earlier observation years. Consequently, while our findings demonstrate contemporaneous spatial associations between building morphology and LST for 2020, this framework cannot establish temporal ordering or causal direction for the observed relationships. Bidirectional relationships or reverse causality may also exist; for example, microclimatic areas experiencing severe LST and persistent heat exposure might actively shape subsequent urban planning decisions, architectural material selections, or local cooling-device (e.g., HVAC) installation densities over time. Future research leveraging multi-temporal urban 3D Level of Detail (LOD) datasets could extend this cross-sectional association model into a longitudinal panel analysis to examine temporal ordering and strengthen causal inference regarding the relevant pathways.

Second, uncertainties in remote-sensing-derived building-height data warrant consideration when interpreting height-related morphometric indicators. The CNBH-10 m dataset achieved nationwide validation statistics of R = 0.77, RMSE = 6.1 m, and MAE = 5.2 m [36]. Validation statistics specific to Hangzhou or the Yangtze River Delta were not reported in the original dataset publication. An independent product comparison found that CNBH performed favorably relative to the other evaluated building-height products, providing supplementary evidence of its relative performance rather than Hangzhou-specific ground-truth validation [64]. However, the comparison identified potential underestimation of high-rise buildings in dense central business districts and overestimation of some low-rise buildings in older urban areas. Because the reference samples represented selected urban areas without exhaustive coverage of all Chinese cities, these deviations may partly reflect the spatial composition of the validation sample. Differences in sensor response, urban morphology, and retrieval methodology may also have contributed to the observed discrepancies. Comparisons involving the fine-grained GABLE dataset similarly highlight localized differences among national building-height products in structurally complex urban environments [65]. Nevertheless, CNBH has been successfully applied to studies of vertical urban growth and building-induced thermal shading across the Yangtze River Delta [52,61]. Related studies in the Yangtze River Delta, Hangzhou, and Shanghai have also cited CNBH in analyses of land-development intensity, functional morphology, and nighttime vitality [66–68]. These applications demonstrate the use of CNBH in regional urban analyses but do not provide Hangzhou-specific ground-truth validation. Suitable local reference data containing building-height information, such as municipal LiDAR, official 3D GIS data, or height-bearing building inventories, were unavailable for the present study. Building footprints alone could support planimetric assessment but cannot independently validate building height. Aggregation to the 100 m × 100 m grid may attenuate pixel-level random noise, but localized systematic bias could still affect height-derived indicators in dense high-rise areas. Future studies should integrate municipal LiDAR, official 3D GIS data, or building inventories containing height information to enable zone-stratified validation and uncertainty propagation.

Third, owing to the lack of spatially consistent building-level data, building-material attributes (e.g., glass curtain walls, concrete, brick, and metal) and construction age were not included in the grid-scale analysis [69]. This omission is relevant to urban-surface thermal behaviour and satellite-derived LST interpretation. Surface emissivity affects Landsat-based surface urban heat-island estimates [70], while solar albedo, thermal admittance, insulation, and material heat-storage properties are associated with energy absorption, storage, and release by building envelopes [71,72]. Observational evidence further indicates that roof albedo and insulation jointly are associated with roof thermal performance [71], whereas modelling studies indicates that the thermal implications of reflective materials vary with urban-canyon geometry [73]. Building density and height heterogeneity can also alter average urban albedo and street-surface temperature [74]. Together, these findings suggest that unmeasured material composition may have introduced local thermal variation relevant to the observed morphology-LST associations. Building age may additionally be associated with differences in envelope performance, building-system efficiency, and operational energy use. For example, modelling for Paris indicated that air-conditioning waste heat could increase local urban air temperature during hot conditions [75]. This result cannot be directly transferred to Hangzhou or interpreted in terms of building age; however, it illustrates that unobserved building attributes and operational characteristics may co-vary with localized thermal heterogeneity. The spatial covariation of these attributes with building morphology could not be quantified using the available data. Their omission may therefore have introduced residual spatial confounding into the estimated associations between building morphology and LST. Accordingly, the results should be interpreted as grid-scale morphology-LST associations rather than independent associations of BCR or BHM. Future work should integrate building inventories, construction-year records, facade and roof material data, and building-energy information, potentially supported by airborne hyperspectral observations and multi-LOD urban building models, to help disentangle geometric, material, and operational associations.

Fourth, regarding scale aggregation and ecological fallacy, analyses were conducted at an aggregated grid-cell scale (100 m × 100 m). Relationships observed at this aggregated spatial grid level may not directly translate to individual building-level thermal processes. The reported associations should therefore be interpreted as grid-level characteristics of aggregated urban-form configurations and cannot be directly generalized to individual buildings. Certain metrics, such as Sky View Factor (SVF), are highly sensitive to building height, spacing, orientation, and street-canyon geometry within each grid cell. Grid aggregation may consequently smooth within-cell heterogeneity, and the estimates may be sensitive to grid size and grid-cell delineation.

Fifth, to address the spatial-resolution limitations of satellite thermal imagery, future studies could leverage UAV-based low-altitude multimodal remote sensing (UAV-LARS) to capture high-resolution 2D/3D building geometry and surface thermal characteristics at the neighborhood scale. When combined with in situ sensors, UAV-based observations could also provide local microclimatic measurements and complement satellite-based grid analyses with building-scale thermal information [18,19]. Integrating these observations with building-level thermal measurements, municipal LiDAR, or BIM data, and testing the stability of findings across multiple spatial resolutions, would help distinguish building-level processes from contextual grid-level patterns. Integrating macro-scale satellite observations with UAV-based microscale diagnostics could provide further insight into physical mechanisms associated with the observed grid-level associations.

## 5. Conclusions

This study integrated 2D landscape metrics and 3D building morphological metrics to examine their joint, grid-level spatial associations with LST along the urban-rural gradient in Hangzhou. Geodetector was used to assess the relative explanatory power and interactions of these indicators for the LST pattern in 2020, while the multi-temporal LST data provided the broader thermal context.

Three main conclusions emerge. First, interactions between 2D and 3D indicators generally provided greater explanatory power for LST spatial heterogeneity than individual factors, with several factor pairs showing nonlinear enhancement. This finding indicates that horizontal land-cover structure and vertical building form should be considered jointly in grid-scale thermal analyses. Second, the relative explanatory power of morphological indicators varied across urban zones. BHM, representing average building height, exhibited the greatest explanatory power in the Core Built-up Area, whereas BCR, representing the proportion of built-up land, showed a stronger association with LST in Suburban Built-up Area. These spatial differences are consistent with zone-specific variations in shading, radiative exposure, surface heat storage, and aerodynamic conditions. Third, these zone-specific associations support differentiated planning responses. Within the Core Built-up Area, spatial optimization strategies should emphasize the three-dimensional arrangement of buildings, the preservation of ventilation corridors, and the integration of blue-green infrastructure. In suburban areas, planning may place greater emphasis on controlling the proportion and continuity of built-up land while maintaining connected blue-green infrastructure. These implications concern grid-level spatial associations and should not be interpreted as direct causal relationships at the individual-building level.

Future research should integrate spatially consistent multi-temporal 3D building data, information on building materials and age [69] and building-level thermal observations. Combining satellite observations with UAV-based low-altitude multimodal remote sensing (UAV-LARS), LiDAR, or BIM data could support neighborhood-scale physical characterization and assessment across multiple spatial resolutions [18,19] and help examine the transferability of the identified associations across cities [76] and different climatic contexts [77].

## Supporting information

S1 File Processed data underlying the reported results. (XLSX)

## References

[1] Yue WZ, Xu JH, Xu LH. Study on the ecological and environmental effects of urban land use based on remote sensing imagery--A case study of urban thermal environment and vegetation index. Acta Ecol Sin. 2006;26(05):1450–60. doi: 10.3321/j.issn:1000-0933.2006.05.021

[2] Kolokotroni M, Giridharan R. Urban heat island intensity in London: An investigation of the impact of physical characteristics on changes in outdoor air temperature during summer. Solar Energy. 2008;82(11):986–98. doi: 10.1016/j.solener.2008.05.004

[3] Saitoh TS, Shimada T, Hoshi H. Modeling and simulation of the Tokyo urban heat island. Atmos Environ. 1996;30(20):3431–42. doi: 10.1016/1352-2310(95)00489-0

[4] Alshayeb MJ, Bindajam AA, Mallick J, Shohan AAA. Integrating fuzzy logic and land use dynamics to delineate urban heat island formation zones and assess land surface temperature changes over three decades: A case study of Asir region, Saudi Arabia. Stoch Environ Res Risk Assess. 2025;39(9):3761–80. doi: 10.1007/s00477-025-03036-7

[5] Magee N, Wendler G, Curtis J. The urban heat island effect at Fairbanks, Alaska. Theor Appl Climatol. 1999;64(1–2):39–47. doi: 10.1007/s007040050109

[6] Mohajerani A, Bakaric J, Jeffrey-Bailey T. The urban heat island effect, its causes, and mitigation, with reference to the thermal properties of asphalt concrete. J Environ Manag. 2017;197:522–38. doi: 10.1016/j.jenvman.2017.03.095 28412623

[7] Yang H, Wu Z, Qiu S, Wu F, Dawson RJ, Ford A, et al. Surface urban heat island variations under the 3-decadal urban expansion in China: Patterns and driving factors. Sustain Cities Soc. 2025;130:106640. doi: 10.1016/j.scs.2025.106640

[8] Peng J, Jia J, Liu Y, Li H, Wu J. Seasonal contrast of the dominant factors for spatial distribution of land surface temperature in urban areas. Remote Sens Environ. 2018;215:255–67. doi: 10.1016/j.rse.2018.06.010

[9] Tran H, Uchihama D, Ochi S, Yasuoka Y. Assessment with satellite data of the urban heat island effects in Asian mega cities. Int J Appl Earth Obs Geoinf. 2006;8(1):34–48. doi: 10.1016/j.jag.2005.05.003

[10] Mirzaei PA. Recent challenges in modeling of urban heat island. Sustain Cities Soc. 2015;19:200–6. doi: 10.1016/j.scs.2015.04.001

[11] Sarkar H. Study of land cover and population density influences on urban heat island in tropical cities by using remote sensing and GIS: A methodological consideration. Third FIG Regional Conference; Jakarta, Indonesia; 2004. p. 3–7.

[12] Cipolla SS, Maglionico M, Semprini G, Villani V, Bonoli A. Green roofs as a strategy for urban heat island mitigation in Bologna (Italy). International Symposium on Greener Cities for More Efficient Ecosystem Services in a Climate Changing World 1215; 2017. p. 295–300.

[13] Ramakreshnan L, Aghamohammadi N. The application of nature-based solutions for urban heat island mitigation in Asia: Progress, challenges, and recommendations. Curr Environ Health Rep. 2024;11(1):4–17. doi: 10.1007/s40572-023-00427-2 38172471

[14] Guo X, Du H, Zhan W, Ji Y, Wang C, Wang C, et al. Global patterns and determinants of year-to-year variations in surface urban heat islands. ISPRS J Photogramm Remote Sens. 2025;223:399–412. doi: 10.1016/j.isprsjprs.2025.03.019

[15] Kalnay E, Cai M. Impact of urbanization and land-use change on climate. Nature. 2003;423(6939):528–31. doi: 10.1038/nature01675 12774119

[16] Coseo P, Larsen L. How factors of land use/land cover, building configuration, and adjacent heat sources and sinks explain Urban Heat Islands in Chicago. Landsc Urban Plan. 2014;125:117–29. doi: 10.1016/j.landurbplan.2014.02.019

[17] Kim Y, Yoo C, Im J. Nighttime satellite land surface temperature for urban applications: Achievements, challenges, and future prospects. GISci Remote Sens. 2025;62(1):2527990. doi: 10.1080/15481603.2025.2527990

[18] Zhong X, Voogt J, Zhan W, Zhang X, Shen Z, Zhao L, et al. UAV-based low-altitude remote sensing in built-environment thermal studies: A review across different spatial scales. Build Environ. 2026;303:114937. doi: 10.1016/j.buildenv.2026.114937

[19] Zhong X, Zhao L, Ren P, Wu R, Zhang X, Shen Z, et al. The role of UAV-derived multimodal images in outdoor thermal comfort observation, evaluation, and improvement: A case study in Guangzhou. Sustain Cities Soc. 2025;131:106723. doi: 10.1016/j.scs.2025.106723

[20] Kotharkar R, Surawar M. Land use, land cover, and population density impact on the formation of canopy urban heat islands through traverse survey in the Nagpur urban area, India. J Urban Plan Dev. 2016;142(1):04015003. doi: 10.1061/(asce)up.1943-5444.0000277

[21] Madanian M, Soffianian AR, Koupai SS, Pourmanafi S, Momeni M. Analyzing the effects of urban expansion on land surface temperature patterns by landscape metrics: A case study of Isfahan city, Iran. Environ Monit Assess. 2018;190(4):189. doi: 10.1007/s10661-018-6564-z 29502210

[22] Shi S, Ji S, Luo Z. Spatial heterogeneity, interaction and multi-scale effects of driving factors of heat island intensity in different urban agglomerations. Sustain Cities Soc. 2025;126:106401. doi: 10.1016/j.scs.2025.106401

[23] Liu X, Ming Y, Liu Y, Yue W, Han G. Influences of landform and urban form factors on urban heat island: Comparative case study between Chengdu and Chongqing. Sci Total Environ. 2022;820:153395. doi: 10.1016/j.scitotenv.2022.153395 35081410

[24] Su H, Han G, Li L, Qin H. The impact of macro-scale urban form on land surface temperature: An empirical study based on climate zone, urban size and industrial structure in China. Sustain Cities Soc. 2021;74:103217. doi: 10.1016/j.scs.2021.103217

[25] Yao R, Wang L, Huang X, Liu Y, Niu Z, Wang S, et al. Long-term trends of surface and canopy layer urban heat island intensity in 272 cities in the mainland of China. Sci Total Environ. 2021;772:145607. doi: 10.1016/j.scitotenv.2021.145607 33770859

[26] Huang C, Liu K, Ma T, Xue H, Wang P, Li L. Analysis of the impact mechanisms and driving factors of urban spatial morphology on urban heat islands. Sci Rep. 2025;15(1):18589. doi: 10.1038/s41598-025-04025-0 40425735 PMC12116756

[27] Oke TR. Review of urban climatology 1968-1973. WMO Tech Note. 1974;134:1–132.

[28] Chen J, Zhan W, Jin S, Han W, Du P, Xia J, et al. Separate and combined impacts of building and tree on urban thermal environment from two- and three-dimensional perspectives. Build Environ. 2021;194:107650. doi: 10.1016/j.buildenv.2021.107650

[29] Hu Y, Dai Z, Guldmann J-M. Modeling the impact of 2D/3D urban indicators on the urban heat island over different seasons: A boosted regression tree approach. J Environ Manag. 2020;266:110424. doi: 10.1016/j.jenvman.2020.110424 32392133

[30] Tian Y, Zhou W, Qian Y, Zheng Z, Yan J. The effect of urban 2D and 3D morphology on air temperature in residential neighborhoods. Landsc Ecol. 2019;34(5):1161–78. doi: 10.1007/s10980-019-00834-7

[31] Tan J, Zheng Y, Tang X, Guo C, Li L, Song G, et al. The urban heat island and its impact on heat waves and human health in Shanghai. Int J Biometeorol. 2010;54(1):75–84. doi: 10.1007/s00484-009-0256-x 19727842

[32] Zheng Q, Xu J, Liu M, Ji K, Zhang Y, Wu S, et al. Mapping the research landscape: A comprehensive bibliometric review of global warming and human health. Int J Climatol. 2025;45(5). doi: 10.1002/joc.8761

[33] Lu L, Fu P, Dewan A, Li Q. Contrasting determinants of land surface temperature in three megacities: Implications to cool tropical metropolitan regions. Sustain Cities Soc. 2023;92:104505. doi: 10.1016/j.scs.2023.104505

[34] Xie ZQ, Du Y, Zeng Y, Gao P, Miao Q. Impact of urban clusters on spatial pattern of extreme high temperature events over Yangtze River Delta. Chin Sci Bull. 2017;62(2/3):233–44. doi: 10.1360/n972016-00643

[35] Center CN. Climate change risk assessment and future scenario projections for major urban areas in China. Beijing: Greenpeace; 2021.

[36] Wu W, Ma J, Banzhaf E, Meadows ME, Yu Z, Guo F, et al. A first Chinese building height estimate at 10 m resolution (CNBH-10 m) using multi-source earth observations and machine learning. Remote Sens Environ. 2023;291:113578. doi: 10.1016/j.rse.2023.113578

[37] Peng J, Ma J, Liu Q, Liu Y, Hu Y, Li Y, et al. Spatial-temporal change of land surface temperature across 285 cities in China: An urban-rural contrast perspective. Sci Total Environ. 2018;635:487–97. doi: 10.1016/j.scitotenv.2018.04.105 29677674

[38] Oke TR. Street design and urban canopy layer climate. Energy Build. 1988;11(1–3):103–13. doi: 10.1016/0378-7788(88)90026-6

[39] Srivanit M, Kazunori H. The influence of urban morphology indicators on summer diurnal range of urban climate in Bangkok metropolitan area, Thailand. Int J Civ Environ Eng. 2011;11(5):34–46.

[40] Wang JF, Xu CD. Geodetector: Principle and prospective. Acta Geogr Sin. 2017;72(1):116–34. doi: 10.11821/dlxb201701010

[41] Ezimand K, Azadbakht M, Aghighi H. Analyzing the effects of 2D and 3D urban structures on LST changes using remotely sensed data. Sustain Cities Soc. 2021;74:103216. doi: 10.1016/j.scs.2021.103216

[42] Luo P, Yu B, Li P, Liang P, Liang Y, Yang L. How 2D and 3D built environments impact urban surface temperature under extreme heat: A study in Chengdu, China. Build Environ. 2023;231:110035. doi: 10.1016/j.buildenv.2023.110035

[43] Zheng B, Myint SW, Fan C. Spatial configuration of anthropogenic land cover impacts on urban warming. Landsc Urban Plan. 2014;130:104–11. doi: 10.1016/j.landurbplan.2014.07.001

[44] Berger C, Rosentreter J, Voltersen M, Baumgart C, Schmullius C, Hese S. Spatio-temporal analysis of the relationship between 2D/3D urban site characteristics and land surface temperature. Remote Sens Environ. 2017;193:225–43. doi: 10.1016/j.rse.2017.02.020

[45] Cai Z, Han G, Chen M. Do water bodies play an important role in the relationship between urban form and land surface temperature? Sustain Cities Soc. 2018;39:487–98. doi: 10.1016/j.scs.2018.02.033

[46] Guo G, Wu Z, Xiao R, Chen Y, Liu X, Zhang X. Impacts of urban biophysical composition on land surface temperature in urban heat island clusters. Landsc Urban Plan. 2015;135:1–10. doi: 10.1016/j.landurbplan.2014.11.007

[47] Arnfield AJ. Two decades of urban climate research: A review of turbulence, exchanges of energy and water, and the urban heat island. Int J Climatol. 2003;23(1):1–26. doi: 10.1002/joc.859

[48] Oke T. The energetic basic of the urban heat island. Q J R Meteorol Soc. 1982;108(455):1–24. doi: 10.1256/smsqj.45501

[49] Voogt JA, Oke TR. Thermal remote sensing of urban climates. Remote Sens Environ. 2003;86(3):370–84. doi: 10.1016/s0034-4257(03)00079-8

[50] Weng Q. Thermal infrared remote sensing for urban climate and environmental studies: Methods, applications, and trends. ISPRS J Photogramm Remote Sens. 2009;64(4):335–44. doi: 10.1016/j.isprsjprs.2009.03.007

[51] Yuan F, Bauer ME. Comparison of impervious surface area and normalized difference vegetation index as indicators of surface urban heat island effects in Landsat imagery. Remote Sens Environ. 2007;106(3):375–86. doi: 10.1016/j.rse.2006.09.003

[52] Qiu L, Ma H, Yuan H, Chen H. Quantify the shading effects on alleviating human thermal stress across different local climate zones in the Yangtze River Delta. Build Environ. 2025;282:113249. doi: 10.1016/j.buildenv.2025.113249

[53] Oke TR. Street design and urban canopy layer climate. Energy Build. 1988;11(1–3):103–13. doi: 10.1016/0378-7788(88)90026-6

[54] Zhou W, Tian Y. Effects of urban three-dimensional morphology on thermal environment: A review. Acta Ecol Sin. 2020;40(2):416–27. doi: 10.5846/stxb201902250353

[55] Alavipanah S, Schreyer J, Haase D, Lakes T, Qureshi S. The effect of multi-dimensional indicators on urban thermal conditions. J Clean Prod. 2018;177:115–23. doi: 10.1016/j.jclepro.2017.12.187

[56] Yuan S, Zhang J, Xu Z, Tang G, Wang C, Chen J. Response of urban heat island effect to building form and its diurnal variation. Geogr Geo-Inf Sci. 2022;38(05):40–6. doi: 10.3969/j.issn.1672-0504.2022.05.006

[57] Li J, Song C, Cao L, Zhu F, Meng X, Wu J. Impacts of landscape structure on surface urban heat islands: A case study of Shanghai, China. Remote Sens Environ. 2011;115(12):3249–63. doi: 10.1016/j.rse.2011.07.008

[58] Massaro E, Schifanella R, Piccardo M, Caporaso L, Taubenböck H, Cescatti A, et al. Spatially-optimized urban greening for reduction of population exposure to land surface temperature extremes. Nat Commun. 2023;14(1):2903. doi: 10.1038/s41467-023-38596-1 37217522 PMC10203342

[59] Zhou W, Wang J, Qian Y, Pickett STA, Li W, Han L. The rapid but “invisible” changes in urban greenspace: A comparative study of nine Chinese cities. Sci Total Environ. 2018;627:1572–84. doi: 10.1016/j.scitotenv.2018.01.335 30857118

[60] Cai H, Xu X. Impacts of built-up area expansion in 2D and 3D on regional surface temperature. Sustainability. 2017;9(10):1862. doi: 10.3390/su9101862

[61] Yin C, Chen R, Xiao X, Qin Y, Meng F, Yao Y, et al. Spatio-temporal evolution of vertical urban growth in China’s Yangtze River Delta from 1990 to 2020. Land Use Policy. 2025;153:107542. doi: 10.1016/j.landusepol.2025.107542

[62] Zheng Z, Zhou W, Yan J, Qian Y, Wang J, Li W. The higher, the cooler? Effects of building height on land surface temperatures in residential areas of Beijing. Phys Chem Earth Parts A/B/C. 2019;110:149–56. doi: 10.1016/j.pce.2019.01.008

[63] Yuan B, Zhou L, Hu F, Wei C. Effects of 2D/3D urban morphology on land surface temperature: Contribution, response, and interaction. Urban Clim. 2024;53:101791. doi: 10.1016/j.uclim.2023.101791

[64] Che Y, Li X, Liu X, Wang Y, Liao W, Zheng X, et al. 3D-GloBFP: The first global three-dimensional building footprint dataset. Earth Syst Sci Data. 2024;16(11):5357–74. doi: 10.5194/essd-16-5357-2024

[65] Sun X, Huang X, Mao Y, Sheng T, Li J, Wang Z, et al. GABLE: A first fine-grained 3D building model of China on a national scale from very high resolution satellite imagery. Remote Sens Environ. 2024;305:114057. doi: 10.1016/j.rse.2024.114057

[66] Shen W, Hu Q, Zhang Z, Niu L. Comprehending the interaction between urban function and morphology at traffic analysis zones scale: The case study from Hangzhou. Geogr J. 2025;191(2):e12620. doi: 10.1111/geoj.12620

[67] Wang X, You L, Wang L. Spatiotemporal coupling characteristics between urban land development intensity and population density from a building-space perspective: A case study of the Yangtze River Delta urban agglomeration. Land. 2025;14(7):1459. doi: 10.3390/land14071459

[68] Xie Q, Cai C, Jiang Y, Zhang H, Wu Z, Xu J. Investigating the performance of SDGSAT-1/GIU and NPP/VIIRS nighttime light data in representing nighttime vitality and its relationship with the built environment: A comparative study in Shanghai, China. Ecol Indic. 2024;160:111945. doi: 10.1016/j.ecolind.2024.111945

[69] Aksoezen M, Daniel M, Hassler U, Kohler N. Building age as an indicator for energy consumption. Energy Build. 2015;87:74–86. doi: 10.1016/j.enbuild.2014.10.074

[70] Chakraborty T, Lee X, Ermida S, Zhan W. On the land emissivity assumption and Landsat-derived surface urban heat islands: A global analysis. Remote Sens Environ. 2021;265:112682. doi: 10.1016/j.rse.2021.112682

[71] Ramamurthy P, Sun T, Rule K, Bou-Zeid E. The joint influence of albedo and insulation on roof performance: An observational study. Energy Build. 2015;93:249–58. doi: 10.1016/j.enbuild.2015.02.040

[72] Santamouris M. Cooling the cities – A review of reflective and green roof mitigation technologies to fight heat island and improve comfort in urban environments. Solar Energy. 2014;103:682–703. doi: 10.1016/j.solener.2012.07.003

[73] Salvati A, Kolokotroni M, Kotopouleas A, Watkins R, Giridharan R, Nikolopoulou M. Impact of reflective materials on urban canyon albedo, outdoor and indoor microclimates. Build Environ. 2022;207:108459. doi: 10.1016/j.buildenv.2021.108459

[74] Yang X, Li Y. The impact of building density and building height heterogeneity on average urban albedo and street surface temperature. Build Environ. 2015;90:146–56. doi: 10.1016/j.buildenv.2015.03.037

[75] de Munck C, Pigeon G, Masson V, Meunier F, Bousquet P, Tréméac B, et al. How much can air conditioning increase air temperatures for a city like Paris, France? Int J Climatol. 2012;33(1):210–27. doi: 10.1002/joc.3415

[76] Lu R, Xu K, Chen R, Chen W, Li F, Lv C. Heat waves in summer 2022 and increasing concern regarding heat waves in general. Atmos Ocean Sci Lett. 2023;16(1):100290. doi: 10.1016/j.aosl.2022.100290

[77] Lin Z, Xu H, Yao X, Yang C, Yang L. Exploring the relationship between thermal environmental factors and land surface temperature of a “furnace city” based on local climate zones. Build Environ. 2023;243:110732. doi: 10.1016/j.buildenv.2023.110732

